# PhytoSelectDBT: A database for the molecular models of anti-diabetic targets docked with bioactive peptides from selected ethno-medicinal plants

**DOI:** 10.6026/97320630019908

**Published:** 2023-09-30

**Authors:** Susanta Roy, Robindra Teron, Raju Nikku Linga

**Affiliations:** 1 Department of Life Science, Assam University - Diphu Campus, Diphu, Karbi Anglong, ASSAM - 782 462; 2 North Eastern Institute of Ayurveda and Folk Medicine Research (NEIAFMR) Pasighat, East Siang District, Arunachal Pradesh - 791102

**Keywords:** Diabetes, ethnomedicinal plants, antidiabetic protein target, antidiabetic plant peptide, molecular docking, diabetes and its complication management

## Abstract

It is of interest to assess the effectiveness of bioactive peptides derived from 41 ethno-medicinal plants, classify them according to their anti-diabetic protein targets (DPP-IV, alpha-amylase, alpha-glucosidase, GRK2, GSK3B, GLP-1R, and AdipoR1), and create a web tool named PhytoSelectDBT by using the top seven peptides per target. If one of the target-based medicinal plant suggestions made by PhytoSelectDBT is unsuccessful, alternative target-based possibilities are presented by PhytoSelectDBT for treating the condition and any other related complications. The results provide a useful resource for the management of type 2 diabetes and emphasize the significance of utilising ethnomedical knowledge for the identification of potent anti-diabetic plants and their peptides. We used molecular docking to investigate interactions between anti-diabetic targets (DPP-IV, alpha-amylase, alpha-glucosidase, GRK2, GSK3B, GLP-1R, and AdipoR1) and projected bioactive peptides from 41 ethnomedicinal plants. All bioactive peptides were cross-checked against several databases to determine their allergenicity, toxicity, and cross-reactivity. The presence of B and T cell epitopes was also examined in all simulated digested bioactive peptides for reference. This data is archived at the PhytoselectDBT database.

## Background:

Diabetes Mellitus (DM) is a lifestyle disease and there is no cure. -Globally, an estimated 422 million adults were living with Diabetes in 2014 II - WHO Report [1]. Type 2 diabetes mellitus (T2DM) accounts for around 90% of all cases of diabetes [2]. For an adult with diabetes in a low-income Indian family, 25 percent of the family income goes to diabetes care [3]. The cost burden is too high in the cases of complications [4]. In remote areas like 'Northeast India', most people do not have access to proper medical facilities. So, people here prefer cheap medical healthcare sources due to the burden of expenditure on diabetes management. Here local ethnomedicinal practitioners with the traditional knowledge of ethnomedicinal plants play an important role in their life. Other than the knowledge of ethnomedicinal plants and disease association, they actually are not aware of what the molecular phytoconstituents that work or what their mechanism of action from antidiabetic plants is. In this scenario and without the knowledge of currently available scientific research conclusions about ethnomedicinal plants and their phyto constituents, there is a high probability that the selection of a wrong medicinal plant based on only current traditional knowledge of ethnomedicine can contribute to disease complications or drug resistance.

The treatment must not only be safe and effective but also improve the quality of life [5]. In 2011 India was home to 61.3 million diabetes patients, according to IDF (International Diabetes Federation) Diabetes Atlas, and by 2030 this number of diabetes patients are predicted to reach 101.2 million [6]. Type 1 diabetes mellitus results when the pancreas fails to produce enough insulin. Type 2 diabetes mellitus (T2D) - is a condition that appears when the body produces inadequate amounts of insulin or the insulin that is produced does not function correctly to control blood glucose level [7]. In India management of this metabolic disorder faces multiple challenges, such as low levels of awareness, scarcity of trained medical and paramedical workers and unaffordability of prescription drugs and services [8]. Novel involvement of existing resources promises to revolutionize the care of diabetic patients in India [8].

Several conventional antidiabetic plant treatments are employed worldwide and are also considered to have less side effects and less toxic than synthetic drug treatments [9]. Although there are medicinal drugs to treat various forms of diabetes mellitus, other complimentary/traditional herbs are used in patients with diabetes [10]. Studies conducted to explore patient preferences have shown that patients prefer less expensive treatment, have fewer side effects, and are more convenient and more effective [10]. As per ancient literature, more than 800 plants are reported to have antidiabetic properties [11]. To control hypoglycemia, ethano pharmacological survey reported more than 1200 plants used in traditional medicine [11]. Indian Materia Medica has mentioned various dravyas effective in Madhumeha [11]. Different ethnic groups have been unconsciously using plants possessing antidiabetic property. Some studies have shown that herbs can delay the progression of diabetic complications, while other studies have shown that some herbs used in diabetes management are not effective [10]. Drugs with the potential to target more metabolic pathways are more promising than those targeting a single pathway, but also correlated with adverse effects are those drugs targeting several pathways [12]. There is a great debate on the optimum risk-benefit profile about therapeutic strategies and treatment of diabetes due to side effects related to existing drugs. Ethnomedicinal plants target multiple pathways due to multiple phytochemicals present in them so there is a need of careful selection of ethnomedicine in ethnomedicinal plant practices. A review study on the toxicity of numerous ethnomedicinal herbs that are also antidiabetic in animal models was published in 1998 by Gupta and Raina. They are Garlic (*Allium sativum*); *Prunus virginiana*; *Sambucus Canadensis* L. [13]; *Vinca rosea*; *Colchicum autumnale*; *Gloriosa superba*; *Matricaria chamomilla*; *Acorus calamus*; *Cassia angustifolia*; *Senna alexandrina* (Synonym: Cassia senna L.); *Rosmarinus officinalis*; *Eucalyptus globulus* (eucalyptus); *Humulus lupulus*; *Glycyrrhiza glabra*; *Capsicum frutescens*; *Aconitum napellus*; *Plantago major* L.; *Diploknema butyracea* [14]. Therefore, medicinal plants should be examined for any potential negative effects before being approved for usage as medications [14].

Data were carefully gathered from a number of sources and published works of literature [15]. We found that 284 different ethnomedical plants are used to treat diabetes in Northeast India. From the list, 39 recognised antidiabetic plants were chosen for this study. Additionally, instead of the plants from the previously mentioned list of antidiabetic plants from northeast India, two ethnomedicinal plants *Murraya paniculata* and *Achyranthes aspera* were chosen which are proved to be antidiabetic in *in vivo* experiments.

Medicinal plants play a major role in controlling Diabetes progression and its associated complications by acting on molecular pathways and key therapeutic targets [16]. The mechanism for phytochemicals' antidiabetic activity could be summed up as: reduction of insulin resistance and increasing of insulin sensitivity, stimulation of insulin secretion from pancreatic cells; stimulation of hepatic glycolysis and glycogenesis; activation of PPARgamma, anti-inflammatory and antioxidant effects, inhibition of alpha-amylase, alpha-glucosidase, and beta-galactosidase, and inhibiting intestinal absorption of glucose [17] etc. After collection of all ethnomedicinal antidiabetic plants of Northeast India we searched the antidiabetic phytochemicals of the 41 selected plants in this study and their mechanism of action from different publication one by one.

Bioactive peptides are specific protein fragments that positively affect physiological functions and human health [18]. These peptides exhibit the following characteristics: (i) targeted high bioactivity; (ii) low toxicity and reduction of the incidence of tissue aggregation in the body; (iii) high structural diversity; and (iv) small size (relative to antibodies) [19]. These properties enable peptides to be applied as therapeutic agents for antidiabetic, antimicrobial, and antioxidant, antithrombotic and antihypertensive functions in human health [20]. Bioactive peptides extracted from plants or microorganisms can control blood glucose levels, reduce insulin resistance and otherwise counter diabetes [21], [22], [23], [24], [25] and [26]. These peptides can be classified according to their antidiabetic actions such as α‐amylase and α-glucosidase inhibitors, GLP-1 receptor agonists and DPP-IV inhibitors [27]. A comprehensive list of Antidiabetic Plant Derived Bioactive Peptides from varied sources can be searched in our work BioDADPep database [28].

Diabetes can affect multiple organ systems in the body over time, and can lead to serious complications [2]. Although understanding of the pathophysiological processes involved in diabetes has improved, with great strides in diabetes control, severe diabetic complications are still faced by patients [29]. If it occurs, it is a serious clinical condition that needs immediate and correct treatment [30]. Complications from diabetes can be classified into microvascular or macrovascular [2]. Hyperglycemia encourages glucose autoxidation to form free radicals. The generation of free radicals beyond the scavenging abilities of endogenous antioxidant defenses results in macro- and microvascular dysfunction [31]. Microvascular complications include nervous system damage (neuropathy), eye damage (retinopathy) and renal system damage (nephropathy) [2]. Macrovascular complications include cardiovascular disease, peripheral vascular disease, and stroke. The latter may lead to bruises or injuries that do not heal, gangrene, and, ultimately, amputation [2]. Therefore, it is of interest to assess the effectiveness of bioactive peptides derived from 41 ethno-medicinal plants (Table 1), classify them according to their anti-diabetic protein targets (DPP-IV, alpha-amylase, alpha-glucosidase, GRK2, GSK3B, GLP-1R, and AdipoR1), and create a web tool PhytoSelectDBT by using the top seven peptides per target.

## Materials and Methods:

## Data collection and organization:

An extensive literature search was undertaken to identify antidiabetic plants of Northeast India from publications. Keywords such as _antidiabetic plants', _antidiabetic phytochemicals', _Diabetes, ethnomedicinal plants', _Northeast India', _Assam', _Nagaland', _Tripura', _Mizoram', _Sikkim', _Manipur', 'Arunachal Pradesh', and _Meghalaya' were used in all combinations in Pubmed search. Ethnic terms such as Garo, Khasi, Bengali, and Assamese were also used with the above terms in all combinations to search literature in Pubmed. The therapeutic mechanism of action for the 41 selected plants was determined, linking their antidiabetic targets with phytoconstituents and/or related knowledge that could be useful for treating Diabetes and its complications, such as Diabetic Cardiomyopathy, Diabetic Retinopathy, Diabetic Nephropathy, and Diabetic Neuropathy. Selected antidiabetic targets are DPP-IV (Dipeptidyl Peptidase IV) inhibitor, Human pancreatic alpha-amylase inhibitor, Intestinal alpha-glucosidase inhibitor, AdipoR1 (Adiponectin Receptor 1) agonist, G Protein Coupled Receptor Kinase Type 2 (GRK2) inhibitor, GSK3beta (Glycogen Synthase Kinase-3 beta) inhibitor, GLP-1R (Glucagon-like Peptide 1 Receptor) agonist. The data is used for plant categorization to develop PhytoSelectDBT. Several animal studies have reported the *in vivo* antidiabetic effects of peptides, but the mechanisms have not been completely elucidated [32]. It is meaningful to enlarge the bioactive peptide database and further explain the mechanisms of these proteins through detailed animal studies [32] especially results out of all bioactive peptides in the selected plants mode.

## Anti-diabetic therapeutic peptide characterization:

The updated steps in the protocol for anti-diabetic therapeutic peptide characterization from selected plant protein are:

[1] Plant protein gastrointestinal digestion

[2] Cross reactivity and Toxicity checks of Peptides

[3] Molecular docking approaches: Anti-diabetic protein target and plant peptide docking

## Plant protein gastrointestinal digestion:

In vitro and *in vivo* studies regarding the bioactivity of peptides has generated strong evidence of their health benefits. Enzymatic hydrolysis has been the process most commonly used for bioactive peptide production [33]. A combination of two to a maximum of three enzymes could be utilized in the hydrolysis simulation of the proteins. Plant protein hydrolysates represent an option for production of bioactive peptides [34]. Predicting possible sites of cleavage for individual proteases is an important task to be completed during drug-design process of peptide therapeutics to improve their stability and availably as a promising drug [34]. For endogenous proteins secreted in the small intestine, only small intestinal digestion was simulated taking into account the reported specificity of trypsin and chymotrypsin only [35].

simulated digestion was conducted using Proteolytic Cleavage tool in CLC Genomics Workbench 12 [36]. A combination of two to a maximum of three enzymes could be utilized in the hydrolysis simulation of the proteins. Protein sequences are digested by the proteolytic enzyme selected (trypsin and chymotrypsin) in below combination to generate peptides: START Trypsin, Trypsin END, Trypsin Trypsin, START Chymotrypsin-low spec., Chymotrypsin-low spec. END, Chymotrypsin-low spec. Chymotrypsin-low spec., START Chymotrypsin-high spec., Chymotrypsin-high spec. END, Chymotrypsin-high spec. Chymotrypsin-high spec., Trypsin Chymotrypsin-low spec., Chymotrypsin-low spec. Trypsin, Trypsin Chymotrypsin-high spec., Chymotrypsin-high spec. Trypsin, Chymotrypsin-high spec. Chymotrypsin-low spec., Chymotrypsin-low spec. Chymotrypsin-high spec. Here, Start: First amino acid of protein sequence; End: Last amino acid of protein sequence.

## Prediction of bioactive peptides:

Peptides identified by plant protein gastrointestinal digestion were screened by PeptideRanker, a tool for the prediction of bioactive peptides based on a novel N-to-1 neural network [37]. Peptides with the score ≥ 0.8 were selected as potential bioactive peptides.

## Peptides cross reactivity and toxicity check:

Peptides cross reactivity and toxicity check studies were performed as per published protocol in BioDADpep Publication [28].

## Molecular docking approaches:

## Anti-diabetic protein targets and plant peptide docking:

Bioactive peptides (predicted by PeptideRanker) molecular docking studies were performed using VLifeMDS 4.6.02032020 software [38] with selected anti-diabetic protein therapeutic targets.

The steps are: (a) Selection of anti-diabetic protein targets, (b) Target proteins (Protein Receptors) preparation (c) Active site Identification, (d) Peptide preparation and (e) Protein-Peptide Docking.

## Selection of anti-diabetic protein targets:

Human Dipeptidyl Peptidase IV (DPP-IV), Alpha-glucosidase, Alpha-amylase, G Protein-Coupled Receptor Kinase 2 (GRK2), GSK-3beta (Glycogen Synthase Kinase-3 beta) and GLP-1R

## Receptor preparation:

The 3D structure of the target protein was reproduced using ModWeb version r230: A Server for Protein Structure Modelling [39]. The crystal structures of the known potent anti-diabetic drug targets were obtained from the Protein Data Bank (PDB). GRK2 protein structures were modeled using the homology modeling program MODELLER [40]. Structures were prepared using UCSF Chimera tool version 1.15: cleaned, fine-tuned and checked in MDweb [41] until all clashes were solved and configurations matched.

## Active site identification:

Protein rigid-body docking (mentioned below) was performed with extracellular region of selected targets. Active sites data retrieved from literature (1) DPP-IV (Dipeptidyl Peptidase IV) inhibitor (PDB: 1NU8) [Chain B], Active site residues in the binding pockets: Glu205, Glu206, Phe357, Tyr662, Arg125, Tyr547, Tyr631, Ser630, Asn709. (2) Human pancreatic alpha-amylase inhibitor (PDB: 4GQR) [Chain A], Active site residues in the binding pocket are Gln63, Trp59, Asp197. (3) Alpha-glucosidase inhibitor (PDB: 3TOP) [Chain A], Active site residues in the binding pocket are Arg1510, Asp1420, Trp1355, Asp1279, Tyr1251, Phe1559, Asp1526, Asp1157. (4) AdipoR1 (Adiponectin Receptor 1) agonist (PDB:6KS0) [Chain A], Active site residues in the binding pocket are Arg267, Phe271, Tyr310, Ser187, His191, Asp208, His337, His341, Cys195, Ala235, Gln335. (5) G Protein Coupled Receptor Kinase Type 2 (GRK2) inhibitor (PDB:3UZS) [Chain A], Active site residues in the binding pocket are Gly201, Phe202, Gly203, Lys220, Met274, Asp278, Ala321, Asp335. (6) GSK3beta (Glycogen Synthase Kinase-3 beta) inhibitor (PDB:1UV5) [Chain A], Active site residues in the binding pocket are Asp133, Val135, Arg141, Gln185. (7) GLP-1R (Glucagon-like Peptide 1 Receptor) agonist (PDB:5NX2) [Chain A], Active site residues in the binding pocket are Leu32, Glu68, Arg121, Tyr152, Arg190, Arg299, Tyr205, Gln234, Thr298, Asn300, Arg380. Extracellular region of target protein active sites was confirmed by TMHMM Server v. 2.0 [42].

## Peptide preparation:

3D structure of 2-4 amino acid peptides were downloaded from Chemsprider [43] and then energetically minimized with VLifeMDS 4.6.02032020. When structures are not available then peptides were predicted using MODPEP [27]. (2) Peptides with more than 5 amino acids were predicted by MODPEP - a fast ab initio structure prediction of linear peptides or disorder proteins [27]. The MODPEP server also offers users an option to refine the generated peptide conformations by a short MD simulation [27]. Model 1 is selected and then energetically minimized with VLifeMDS 4.6.02032020.

## Protein-peptide docking:

For Docking Lenovo computer, i5 equivalent processor (AMD A9 7th Gen) with Windows 10 operating system is used. Peptide screening was performed with selection of appropriate protein structure. Batch docking simulation was done using GRIP batch docking to generate dock score and interactions.

## Docking:

Step 1: Selected option Biopredicta Tools>>Docking>>Grip. Step 2: Selected the protein file to be used for docking. Step 3: Specified the cavity for docking either by selecting the pre saved co-crystal ligand or selecting the cavity number based on residues of active site. Step 4: Selected the folder of Ligands and to save docking results. Step 5: Selected docking parameters and clicked ok, checked the docking job in Task manager. After docking simulation, the best docked conformer of each peptide/ligand was checked for interactions with receptor like hydrogen bonding and other interactions using LigPlot+. LigPlot+ is a successor to original LIGPLOT program for automatic generation of 2D ligand-protein interaction diagrams [44]. LigPlot+ runs from an intuitive java interface which allows on-screen editing of the plots via mouse click-and-drag operations.

## Problem with ethno-medicinal plant practices:

Type 2 Diabetes treatment should be prioritized according to existing evidence [45]. Knowledge of cautiously using ethno-medicinal Plants in Diabetes management is very important using the latest scientific findings so that it is not only useful in Diabetes management but also useful in the management of Diabetes complications. Due to the burden of disease management, people with Type 2 Diabetes prefer ethno-medicinal plants although preclinical/clinical trial data with human subjects are limited and preliminary.

[1] _Multiple phytochemicals' - _multiple target' - _Single plant'.

[2] Phyto-peptides are not well characterized based on mechanism of action on multiple on and off anti-diabetic targets.

[3] There is a high probability that the selection of a wrong medicinal plant-based on traditional knowledge can contribute to disease complications or drug resistance.

So, a tool named PhytoSelectDBT version 2.0 is developed to encounter these problems using functional peptide classification of anti-diabetic plants based on Diabetes and its complication.

## Results and Discussion:

PhytoSelectDBT have valuable information for traditional ethnomedical practitioners to search and select anti-diabetic plants cautiously through (A) Find Ethno-medicinal Plants based on Diabetic Conditions [by _Diabetes', _Diabetic Cardiomyopathy', _Diabetic Nephropathy', _Diabetic Neuropathy', _Diabetic Retinopathy', _Hypertension', 'Obesity] and (B) Change Therapeutic Regime Based on Anti-diabetic Target (s) (by _DPP-IV (Dipeptidyl Peptidase IV) inhibitor', _Human pancreatic alpha-amylase inhibitor', _Intestinal alpha-glucosidase inhibitor', _AdipoR1 (Adiponectin Receptor 1) agonist', _G Protein Coupled Receptor Kinase Type 2 (GRK2) inhibitor', _GSK3beta (Glycogen Synthase Kinase-3 beta) inhibitor' and _GLP-1R (Glucagon-like Peptide 1 Receptor) agonist)'.

Scientists can search presented plant data through (A) and (B), strengthen the confirmation and addition of data to improve ethno-medicinal practices and reduce healthcare burden. After selecting desired check box (s), clicking on _Filter' button will retrieve results with one or more plant name (s). Clicking on -Read More II hyperlink in the search resulted plant name (s)/Images (s) will pop-up the page with following details: (1) Image, (2) Plant name, (3) English name, (4) Local name, (5) Family, (6) Plant part used, (7) Uses/Preparation, (8) Found in the Region/Northeast State, (9) Tribe using the plant, (10) Diabetes Type, (11) Potential toxicity problems, (12) Hyperglycemia. And further whether or not can be used in: (13) Diabetic cardiomyopathy, (14) Diabetic retinopathy, (15) Diabetic nephropathy, (16) Diabetic neuropathy, (17) Obesity, (18) Anti-diabetic targets vs the plant connection (19) In silico, in vitro and *in vivo* experimental Data, (20) Anti-diabetic phytoconstituent (s) and their mechanism of action are also included with (21) References. Input text box is also available (above 'Filter' button) to search PhytoSelectDBT anti-diabetic plant data. PhytoSelectDBT recommended Plants in top search results by protein target useful in Diabetes and its complication management prioritized by best bioactive peptides are shown in Table 2. If one target-based medicinal plant recommendation using PhytoSelectDBT is ineffective, other options for managing disease and associated complications (shown inTable 3,Table 4) are provided by PhytoSelectDBT.

We utilized systematic bioinformatics methods to understand and predict the (mechanism of action of) anti-diabetic peptides. All predicted bioactive Peptides from forty-one plant species were screened against seven anti-diabetic targets. The plant species screened in this study have previously been tested against diabetes in animal models. In this research, we found bioactive anti-diabetic peptides in all selected plants against all selected anti-diabetic human therapeutic targets. After docking simulation, the best docked conformer of each peptide was checked for various interactions with receptor like hydrogen bonding, hydrophobic bonding interaction. Docking of each bioactive peptide resulted in 10 conformations. The docking results were analysed and a pose was selected based on lowest binding energy and H-bond interactions. The peptide forming most stable peptide-receptor complex is the one which is having minimum dock score.

H-bond with active side residues in protein cavity were considered to be vital for the selective therapeutic interactions, interaction stability and selective therapeutic agonistic or antagonistic effect, resulting in higher activity of bioactive peptides. So, in further analysis bioactive peptides those formed H-bond inside the selected target protein cavity with known active site residues interactions are preferred to understand their binding modes. Several hydrogen bonds mediate the interactions between the peptides and protein targets as previously reported. Peptides cross-reactivity check revealed that 54 of our bioactive peptides matched with AHTPDB, BioPepDB, SATPdb, BioDADPep and IEDB (see excel file in website: https://omicsbase.com/PhytoselectDBT/bioactive-peptides-and-iedb-database/).

## Conclusion:

PhytoSelectDBT is useful for understanding multifunctional therapeutic applications and their molecular mechanisms of the anti-diabetic effects. Data will be of help in the understanding the nature of bioactive peptides, their structural properties required for the selection of plants in management of diabetes and its complications; and for designing new anti-diabetic formulations with improved accuracy and precision. However, extensive pharmacology and toxicological studies in animal and human models are further warranted.

## Funding:

No funding sources

## Figures and Tables

**Table 1:** List of selected ethnomedicinal antidiabetic plants, ranked & prioritized in PhytoSelectDBT for diabetes management.

**Sl. No.**	**Scientific name**	**Genus**	**Family**
1	*Abutilon indicum*(L.) Sweet	Abutilon	Malvaceae
2	*Acacia nilotica*(L.) Delile	Acacia	Leguminosae
3	*Achyranthes aspera*L.	Achyranthes	Amaranthaceae
4	*Adiantum capillus-veneris* L.	Adiantum	Pteridaceae
5	*Albizia procera*(Roxb.) Benth.	Albizia	Leguminosae
6	*Annona squamosa*L.	Annona	Annonaceae
7	*Areca catechu*L.	Areca	Arecaceae
8	*Brucea javanica*(L.) Merr.	Brucea	Simaroubaceae
9	*Ficus benghalensis*L.	Ficus	Moraceae
10	*Ficus racemose*L.	Ficus	Moraceae
11	*Ficus religiosa*L.	Ficus	Moraceae
12	*Flacourtia jangomas*(Lour.) Raeusch.	Flacourtia	Salicaceae
13	*Gymnema sylvestre* Retz.) R.Br.	Gymnema	Apocynaceae
14	*Hemidesmus indicus*(L.) R.	Hemidesmus	Apocynaceae
15	*Ipomoea aquatica*Forssk.	Ipomoea	Convolvulaceae
16	*Oroxylum indicum*(L.) Kurz	Oroxylum	Bignoniaceae
17	Phlogacanthus thyrsiflorus Nees	Phlogacanthus	Acanthaceae
18	*Phyllanthus niruri*L.	Phyllanthus	Phyllanthaceae
19	Senna sophera (L.) Roxb.	Senna	Leguminosae
20	*Smilax lanceifolia Roxb.*	Smilax	Smilacaceae
21	*Swertia chirayita*(Roxb.) Buch. -Ham.	Swertia	Gentianaceae
22	*Tabernaemontana divaricate*(L.) R.Br.	Tabernaemontana	Apocynaceae
23	*Terminalia arjuna*(Roxb. ex DC.) Wight and Arn.	Terminalia	Combretaceae
24	*Tinospora crispa*(L.) Hook.	Tinospora	Menispermaceae
25	*Tinospora sinensis*(Lour.) Merr.	Tinospora	Menispermaceae
26	*Aegle marmelos*(L.) Corrˆea	Aegle	Rutaceae
27	*Berberis aristata*DC.	Berberis	Berberidaceae
28	*Bidens pilosa*L.	Bidens	Compositae
29	*Biophytum sensitivum*(L.) DC.	Biophytum	Oxalidaceae
30	*Cassia fistula*L.	Cassia	Leguminosae
31	*Ichnocarpus frutescens*(L.) W.T. Aiton	Ichnocarpus	Apocynaceae
32	*Murraya paniculata*(L.) Jack	Murraya	Rutaceae
33	*Ocimum americanum*L.	Ocimum	Lamiaceae
34	*Ocimum tenuiflorum*L.	Ocimum	Lamiaceae
35	*Terminalia chebula*Retz.	Terminalia	Combretaceae
36	*Urtica dioica*L.	Urtica	Urticaceae
37	*Ficus hispida*L.f.	Ficus	Moraceae
38	*Panax pseudoginseng*Wall.	Panax	Araliaceae
39	*Senna alata*(L.) Roxb.	Senna	Leguminosae
40	*Phyllanthus emblica*L.	Phyllanthus	Phyllanthaceae
41	*Lantana camara*L.	Lantana	Verbenaceae

**Table 2:** Top peptides against anti-diabetic targets and their interactions is shown. Based on top docking score per peptide per anti-diabetic target selected anti-diabetic plants are ranked & prioritized in PhytoSelectDBT.

**Peptides**	**Plants**	**Anti-diabetic Targets**	**Dock score**	**Formed Hydrogen bond after docking**	**Formed Hydrophobic contacts after docking**	**Matching H-bond interactions found with known active site residues after docking**	**Matching Hydrophobic interactions found with known active site residues after docking**
RRPWPIH	*Biophytum sensitivum* (L.) DC.; Brucea javanica (L.) Merr.	DPP-IV	-75.2986	Arg125, Tyr752, Lys554	Trp629, Tyr547, Gly741, Trp627, His740, Asn710	Arg125	Tyr547
ISPSSFPL	*Annona squamosa* L.	alpha-amylase	-76.841	His305, Gln63, Lys200	Trp59, Asp197, Ile235, Gly309, Trp58, Leu165, Glu233, Gly306, Tyr151, Arg195, Ala198, Ile51, Leu162, Asp300, Gly308, Ala310	Gln63	Trp59, Asp197
YPW	*Ficus racemosa* L.; *Ficus religiosa* L.	alpha-glucosidase	-63.3361	Asp1526	Trp1369, Trp1355, Tyr1251, Thr1586, Gln1561, Thr1589	Asp1526	Trp1355, Tyr1251
CR	*Urtica dioica* L.; *Terminalia chebula Retz*.; *Ocimum tenuiflorum* L.; *Murraya paniculata* (L.) Jackic; *Ichnocarpus frutescens* (L.) W.T.Aiton_ proteol; *Cassia fistula* L. cle; *Biophytum sensitivum* (L.) DC.; *Bidens pilosa* L.; *Aegle marmelos* (L.) Corrêa; *Senna alata* (L.) Roxb.; *Phyllanthus emblica* L.; *Lantana camara* L.; *Abutilon indicum* (L.) Sweet; *Achyranthes aspera* L.; *Adiantum capillus-veneris* L.; *Albizia procera* (Roxb.) Benth.; *Annona squamosa* L.; *Brucea javanica* (L.) Merr.; *Ficus benghalensis* L.; *Ficus racemosa* L.; *Ficus religiosa* L.; *Flacourtia jangomas* (Lour.) Raeusch.; *Gymnema sylvestre* (Retz.) R.Br.; *Hemidesmus indicus* (L.) R.; *Ipomoea aquatica Forssk*.; *Oroxylum indicum* (L.) Kurz; *Phyllanthus niruri* L.; *Senna sophera* (L.) Roxb.; *Tabernaemontana divaricata* (L.) R.Br.; *Terminalia arjuna* (Roxb. ex; *Tinospora crispa* (L.) Hook.; *Tinospora sinensis* (Lour.) Merr.	AdipoR1	-51.0052	Arg267, Ala259	Asp106, His337, His191, Tyr209, Glu134, Asn107, Tyr317, Leu110, His114, Thr133, Tyr194, Ser205	Arg267	His191, His337
DGNF	*Annona squamosa* L.	GLP-1R	-66.2874	Gln234, Tyr241, Ala298	Leu384, Glu387, Arg310, Ile313, Leu314, Ala368, Met233, Val237, Trp306, Ile309, Val365, Leu388	Arg190	Tyr152
VLPPIF	*Ficus racemosa* L.; *Ficus religiosa* L.	GSK3B	-73.7042	Val135	Asn64, Leu188, Ile62, Gly63, Ala83, Tyr134, Asp133, Thr138, Gln185	Val135	Asp133, Gln185
WPNYR	*Annona squamosa* L.; *Ficus racemosa* L.; *Ficus religiosa* L.	GRK2	-72.9902	Asp278, Asp484, Ala321	Tyr281, Ala482, Asn275, Arg195, Leu324	Asp278, Ala321	

**Table 3:** List of PhytoSelectDBT recommended plants details based on the lowest binding energy docking scores of plant peptides against anti-diabetic targets and literature review data that is useful in anti-diabetic plant selection.

***Plant Name***	***Anti-diabetic Targets***	***Diabetes Complications***
Urtica dioica L.	Human Pancreatic alpha-amylase [46]; intestinal α-glucosidase [46]	Diabetic Nephropathy [47]; Diabetic Neuropathy [48]; Hypertension [49], [50]
Terminalia chebula Retz.	Human Pancreatic alpha-amylase [51]; intestinal α-glucosidase [52]; DPP-IV [53]	Diabetic Retinopathy [54]; Diabetic Nephropathy [55]; Diabetic Neuropathy [54]; Hypertension [56]; Obesity [57]
Ocimum tenuiflorum L.	Human Pancreatic alpha-amylase [58]; DPP-IV [59]	Diabetic Retinopathy [60]; Hypertension [61]; Obesity [62]
*Murraya paniculata* (L.) Jackic	Intestinal α-glucosidase [63]	Diabetic Nephropathy [64]; Hypertension [65]
Ichnocarpus frutescens (L.) W.T.Aiton	Intestinal α-glucosidase [66]	Diabetic Cardiomyopathy [67], [68]; Diabetic Neuropathy [69]; Obesity [70], [71]
Cassia fistula L.	Human Pancreatic alpha-amylase [58]	Diabetic Nephropathy [72]
Bidens pilosa L.	Human Pancreatic alpha-amylase [73]; intestinal α-glucosidase [74]	Hypertension [75]; Obesity [76]
Aegle marmelos (L.) Corrêa	Human Pancreatic alpha-amylase [77]; intestinal α-glucosidase [78]; DPP-IV [79]	Diabetic Cardiomyopathy [80]; Diabetic Nephropathy [81]; Hypertension [82]; Obesity [83]
Senna alata (L.) Roxb.	Human Pancreatic alpha-amylase [84]; intestinal α-glucosidase [84]	-
Phyllanthus emblica L.	Human Pancreatic alpha-amylase [85]; intestinal α-glucosidase [85]; DPP-IV [86]	Diabetic Cardiomyopathy [87]; Diabetic Retinopathy [54]; Diabetic Nephropathy [88]; Diabetic Neuropathy [89]; Hypertension [90]; Obesity [91]
Lantana camara L.	Human Pancreatic alpha-amylase [92], [93]; intestinal α-glucosidase [93]	Hypertension [94]
Abutilon indicum (L.) Sweet	Human Pancreatic alpha-amylase [95]; intestinal α-glucosidase [95]	Diabetic Nephropathy [96]; Diabetic Neuropathy [97]
*Achyranthes aspera* L.	Human Pancreatic alpha-amylase [98]	Hypertension [99]; Obesity [100], [101]
Adiantum capillus-veneris L.	Human Pancreatic alpha-amylase [102]; intestinal α-glucosidase [102]	Obesity [102]
Albizia procera (Roxb.) Benth.	Human Pancreatic alpha-amylase [103]; intestinal α-glucosidase [103]	-
Annona squamosa L.	Human Pancreatic alpha-amylase [104]; Intestinal α-glucosidase [105]; DPP-IV [106]	Hypertension [107]
Brucea javanica (L.) Merr.	Intestinal α-glucosidase [108]	-
Ficus benghalensis L.	Human Pancreatic alpha-amylase [109]; intestinal α-glucosidase [109]	Obesity [102]
Ficus racemosa L.	Human Pancreatic alpha-amylase [85]; intestinal α-glucosidase [85]	Diabetic Cardiomyopathy [110]; Diabetic Nephropathy [110]; Diabetic Neuropathy [111]; Obesity [112]
Ficus religiosa L.	Human Pancreatic alpha-amylase [113]; DPP-IV [114]	-
Gymnema sylvestre (Retz.) R.Br.	Human Pancreatic alpha-amylase [115]; intestinal α-glucosidase [116]; DPP-IV [86]	Diabetic Retinopathy [117]; Diabetic Neuropathy [118]; Hypertension [119]; Obesity [120]
Ipomoea aquatica Forssk.	Intestinal α-glucosidase [121]	-
Oroxylum indicum (L.) Kurz	Human Pancreatic alpha-amylase [122]; Intestinal α-glucosidase [122]	Diabetic Nephropathy [123]; Obesity [124]
Phyllanthus niruri L.	Intestinal α-glucosidase [125]; DPP-IV [126]	Diabetic Neuropathy [97]; Hypertension [127]
Terminalia arjuna (Roxb. ex	Human Pancreatic alpha-amylase [77]; DPP-IV [86]	Diabetic Cardiomyopathy [128]; Diabetic Nephropathy [129]; Hypertension [130], [131]
Tinospora crispa (L.) Hook.	Human Pancreatic alpha-amylase [132]; intestinal α-glucosidase [132]; DPP-IV [114]	Hypertension [133], [134]
Tinospora sinensis (Lour.) Merr.	Human Pancreatic alpha-amylase [135]; Intestinal α-glucosidase [135]	-

**Table 4:** Toxicity Prediction with ToxinPred [141] of bioactive peptides revealed the below results.

**UniProtKB Entry Name**	**Peptide**	**Protein Names**	**(Start - Stop)**	**Organism**	**ToxinPred Prediction of the Peptide**
Q9S705_URTDI	CCSIW	Agglutinin isolectin VII (Fragment)	(40 - 44)	*Urtica dioica*	Toxin
Q9S7B3_URTDI	CCSIW	Agglutinin isolectin V (Fragment)	(40 - 44)	*Urtica dioica*	Toxin
Q9SYR2_URTDI	CCSIW	Putative agglutinin isolectin III (Fragment)	(40 - 44)	*Urtica dioica*	Toxin
Q9SYR5_URTDI	CCSIW	Agglutinin isolectin VI (Fragment)	(40 - 44)	*Urtica dioica*	Toxin
Q9ZP51_URTDI	CCSIW	Agglutinin isolectin IV (Fragment)	(40 - 44)	*Urtica dioica*	Toxin
AGI_URTDI	CCSIW	Lectin/endochitinase 1	(40 - 44)	*Urtica dioica*	Toxin
Q9S7K1_URTDI	CCSIW	Agglutinin isolectin I (Fragment)	(40 - 44)	*Urtica dioica*	Toxin
Q9S7W3_URTDI	CCSIW	Agglutinin isolectin I (Fragment)	(40 - 44)	*Urtica dioica*	Toxin
Q9S7C2_URTDI	CCSIW	Agglutinin isolectin II (Fragment)	(41 - 45)	*Urtica dioica*	Toxin
A0A2Z4LID3_PHYNR	CQCCF	Maturase K (Fragment)	(119 - 123)	*Phyllanthus niruri*	Toxin
Q49C39_PHYNR	CQCCF	Maturase K	(306 - 310)	*Phyllanthus niruri*	Toxin
A0A0N9XTR6_9ROSA	GTSCCF	NAD(P)H-quinone oxidoreductase subunit K, chloroplastic	(68 - 73)	*Ficus racemosa*	Toxin
A0A1Q1MNJ8_9ROSA	GTSCCF	NAD(P)H-quinone oxidoreductase subunit K, chloroplastic	(98 - 103)	Ficus religiosa	Toxin
A0A0P0JQQ9_BRAJU	GTSCCF	NAD(P)H-quinone oxidoreductase subunit K, chloroplastic	(40 - 45)	Brassica juncea	Toxin
A0A1Q1MNI1_9ROSA	IACCF	Photosystem II CP43 reaction center protein	(285 - 289)	Ficus religiosa	Toxin
A0A0N7H9X7_9ROSA	IACCF	Photosystem II CP43 reaction center protein	(295 - 299)	*Ficus racemosa*	Toxin
A0A0P0KE14_BRAJU	IACCF	Photosystem II CP43 reaction center protein	(285 - 289)	Brassica juncea	Toxin
